# Noninvasive evaluation of pulmonary artery stiffness in heart failure patients via cardiovascular magnetic resonance

**DOI:** 10.1038/s41598-023-49325-5

**Published:** 2023-12-19

**Authors:** Xuewen Hou, Djawid Hashemi, Jennifer Erley, Marthe Neye, Paulius Bucius, Radu Tanacli, Titus Kühne, Marcus Kelm, Laura Motzkus, Moritz Blum, Frank Edelmann, Wolfgang M. Kuebler, Burkert Pieske, Hans-Dirk Düngen, Andreas Schuster, Lukas Stoiber, Sebastian Kelle

**Affiliations:** 1 Department of Cardiology, Angiology and Intensive Care Medicine, Deutsches Herzzentrum der Charité, Augustenburger Platz 1, 13353 Berlin, Germany; 2 Charité-Universitätsmedizin Berlin, Corporate Member of Freie Universität Berlin and Humboldt-Universität zu Berlin, Charitéplatz 1, 10117 Berlin, Germany; 3 DZHK (German Center for Cardiovascular Research), Partner Site Berlin, Berlin, Germany; 4 Berlin Institute of Health at Charité-Universitätsmedizin Berlin, BIH Biomedical Innovation Academy, BIH Charité Digital Clinician Scientist Program, Charitéplatz 1, 10117 Berlin, Germany; 5 Department of Diagnostic and Interventional Radiology and Nuclear Medicine, University Medical Center Hamburg-Eppendorf, Hamburg, Germany; 6 Department of Cardiology, Medical Academy, Lithuanian University of Health Sciences, Kaunas, Lithuania; 7 Deutsches Herzzentrum der Charité, Institute of Computer-Assisted Cardiovascular Medicine, Augustenburger Platz 1, 13353 Berlin, Germany; 8 Department of Congenital Heart Disease-Pediatric Cardiology, Deutsches Herzzentrum der Charité, Augustenburger Platz 1, 13353 Berlin, Germany; 9 Brookdale Department of Geriatrics and Palliative Medicine, Icahn School of Medicine at Mount Sinai, New York, NY USA; 10 Department of Cardiology and Pneumology, University Medical Center Göttingen, Georg-August University, Göttingen, Germany; 11 Institute of Physiology, Charité-Universitätsmedizin Berlin, Berlin, Germany; 12 DZHK (German Center for Cardiovascular Research), Partner Site Göttingen, Göttingen, Germany; 13 Royal Brompton Hospital, Guy’s and St Thomas’ National Health Service Foundation Trust, London, UK

**Keywords:** Cardiology, Medical imaging, Cardiovascular diseases, Heart failure

## Abstract

Heart failure (HF) presents manifestations in both cardiac and vascular abnormalities. Pulmonary hypertension (PH) is prevalent in up 50% of HF patients. While pulmonary arterial hypertension (PAH) is closely associated with pulmonary artery (PA) stiffness, the association of HF caused, post-capillary PH and PA stiffness is unknown. We aimed to assess and compare PA stiffness and blood flow hemodynamics noninvasively across HF entities and control subjects without HF using CMR. We analyzed data of a prospectively conducted study with 74 adults, including 55 patients with HF across the spectrum (20 HF with preserved ejection fraction [HFpEF], 18 HF with mildly-reduced ejection fraction [HFmrEF] and 17 HF with reduced ejection fraction [HFrEF]) as well as 19 control subjects without HF. PA stiffness was defined as reduced vascular compliance, indicated primarily by the relative area change (RAC), altered flow hemodynamics were detected by increased flow velocities, mainly by pulse wave velocity (PWV). Correlations between the variables were explored using correlation and linear regression analysis. PA stiffness was significantly increased in HF patients compared to controls (RAC 30.92 ± 8.47 vs. 50.08 ± 9.08%, p < 0.001). PA blood flow parameters were significantly altered in HF patients (PWV 3.03 ± 0.53 vs. 2.11 ± 0.48, p < 0.001). These results were consistent in all three HF groups (HFrEF, HFmrEF and HFpEF) compared to the control group. Furthermore, PA stiffness was associated with higher NT-proBNP levels and a reduced functional status. PA stiffness can be assessed non-invasively by CMR. PA stiffness is increased in HFrEF, HFmrEF and HFpEF patients when compared to control subjects.

**Trial registration** The study was registered at the German Clinical Trials Register (DRKS, registration number: DRKS00015615).

## Background

Heart failure (HF) is a systemic syndrome affecting both the heart and the vascular system with an increasing prevalence^1,2^. While established treatment strategies have improved the prognosis in patients with HF with reduced ejection fraction (HFrEF) and recent studies also showed first effective therapies in patients with HF with mildly reduced ejection fraction (HFmrEF) or preserved ejection fraction (HFpEF), the prognosis of patients with HF remains poor and is heterogeneous across the population^1,3,4^. A major pillar of HF therapy is the pharmacological reduction of the increased afterload and vascular resistance, primarily driven by the activation of the renin–angiotensin–aldosterone system (RAAS)^1^. Pulmonary hypertension is highly prevalent among patients with HF, present in approximately 50% of those with HFrEF or HFpEF, and it is associated with worst outcomes^5–8^. PA stiffness is a manifestation of PH, the feasibility to detect PA stiffness by CMR and its predictive value for an early diagnosis pulmonary arterial hypertension (PAH) has already been shown^9–11^. The prognostic role of PA stiffness in HF and post-capillary PH is yet unknown^12–15^.

Currently, right-heart catheterization is the reference standard method to evaluate PA stiffness and flow hemodynamics. Echocardiographic attempts to assess PA stiffness and hemodynamic flow parameters are limited by frequent suboptimal acoustic windows. Cardiovascular magnetic resonance imaging (CMR) is a comprehensive technique, increasingly accessible and provides not only high resolution information on functional cardiac parameters but also details on vascular contraction and blood flow characteristics. In particular, the phase contrast magnetic resonance (PCMR) technique allows for structural and functional evaluation of blood vessels.

CMR-based assessment of PA stiffness and flow hemodynamics has been demonstrated to be accurate in patients with chronic obstructive pulmonary disease and PAH^9,11,16–18^. The applicability in a high-risk group for post-capillary PH with HF has not been shown.

In this study, we aimed to (a) assess PA stiffness and parameters of pulmonary hemodynamics by CMR in both patients with HF and subjects without HF, to (b) analyze their association with symptom burden, and to (c) compare the results between HF and control subjects groups.

## Methods

### Study population and design

This study was a prospective study conducted at two centers in Berlin, Germany, namely at the Charité—University Medicine Berlin and the German Heart Centre Berlin, between 2017 and 2018. Its rationale and design of the study as well as data and primary results of other research questions from the described patient cohort have been previously published^19–23^.

Briefly, subjects with a history of HF and an age of at least 45 years could be included. The initial diagnosis of HF had to be made at least 30 days ago; patients were required to be in a stable state with no changes in their HF medication and no HF hospitalization within the previous 7 days. HFrEF was defined by LVEF < 40%, HFmrEF by an increased NT-proBNP (> 220 pg/mL) and LVEF ≥ 40% and < 50% as well as HFpEF by an increased NT-proBNP (> 220 pg/mL) and LVEF ≥ 50% at the time of study inclusion^19^. We did not distinguish between the causes of HF for recruiting patients^19^.

Additionally, we recruited subjects without HF or advanced cardiovascular (CV) diseases as controls. All studies complied with the Declaration of Helsinki, the protocols were approved by the responsible ethics committees, and all patients gave written informed consent. The study was registered at the German Clinical Trials Register (DRKS, registration number: DRKS00015615). Detailed inclusion and exclusion criteria are listed on the webpage of the DRKS.

### CMR protocol

All examinations were performed in the supine position using a 1.5 T MR scanner (Achieva, Philips, Best, The Netherlands). Short- and long-axis cine images were acquired during breath holds of 10–15 s with a standard balanced steady-state free precession sequence. Main PA cross-sectional images were obtained for blood flow quantification by using a 2D phase-contrast acquisition with through-plane velocity encoding perpendicular to the pulmonary artery trunk, with imaging parameters as follows: 35 phases per cardiac cycle; flip angle 15 degrees, slice thickness 8 mm, field of view 340 × 400 mm, temporal resolution 25 ms, velocity encoding 200 cm/s (cross-sectional image of the PA using phase-contrast through-plane sequences illustrated in supplementary material S1).

### CMR image analysis

**Figure 1:**
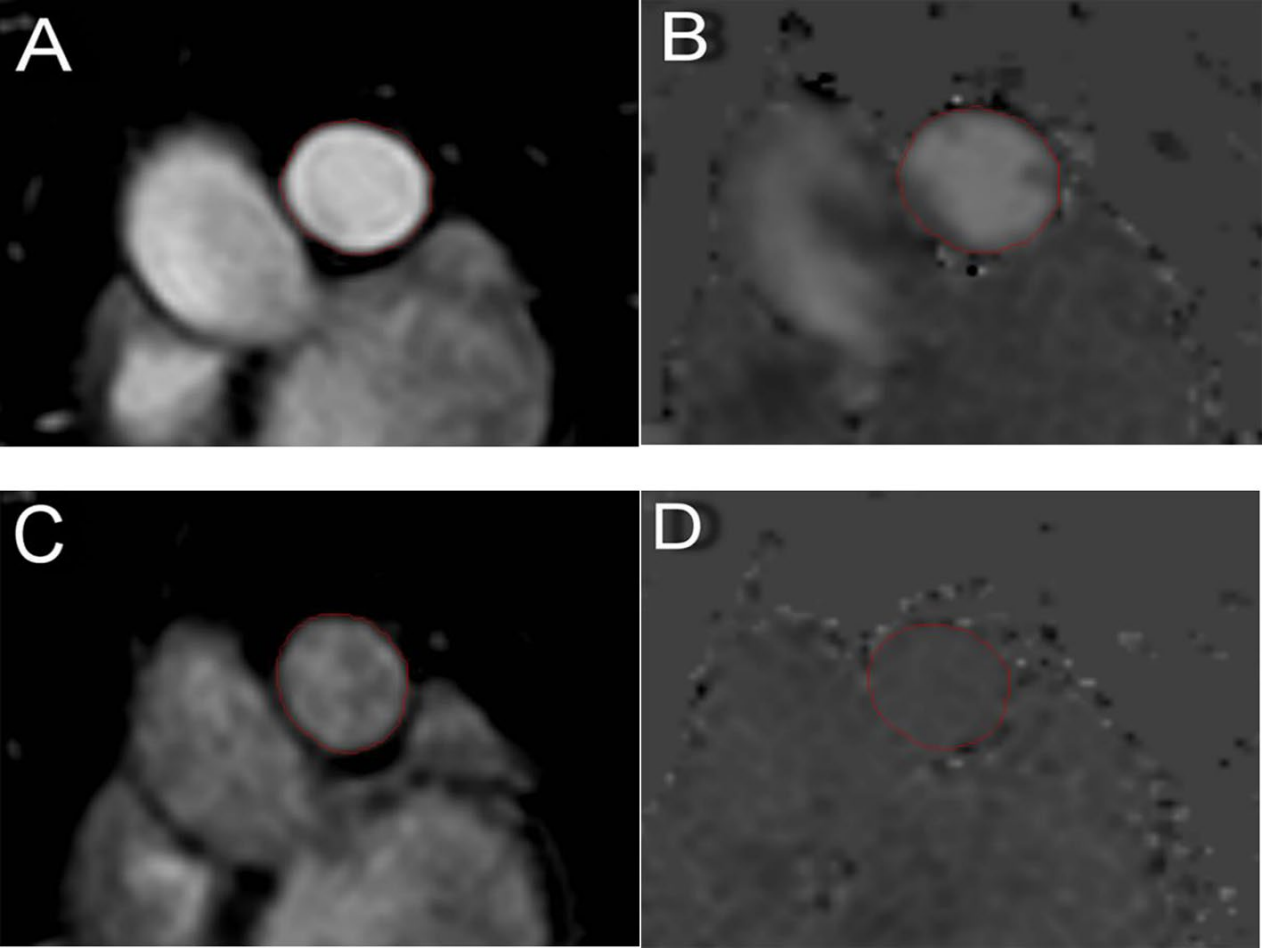
PA cross-sectional MR images. (**A**–**D**) PA cross-sectional phase-contrast MR image acquisition perpendicular to the pulmonary trunk. Cross-sectional PA areas were measured by (**A**) magnitude and (**B**) velocity images in end-systole and (**C**) magnitude and (**D**) velocity images in end-diastole. Vessel contours (red circle) were traced throughout the entire cardiac cycle. *PA* pulmonary artery.

**Figure 2:**
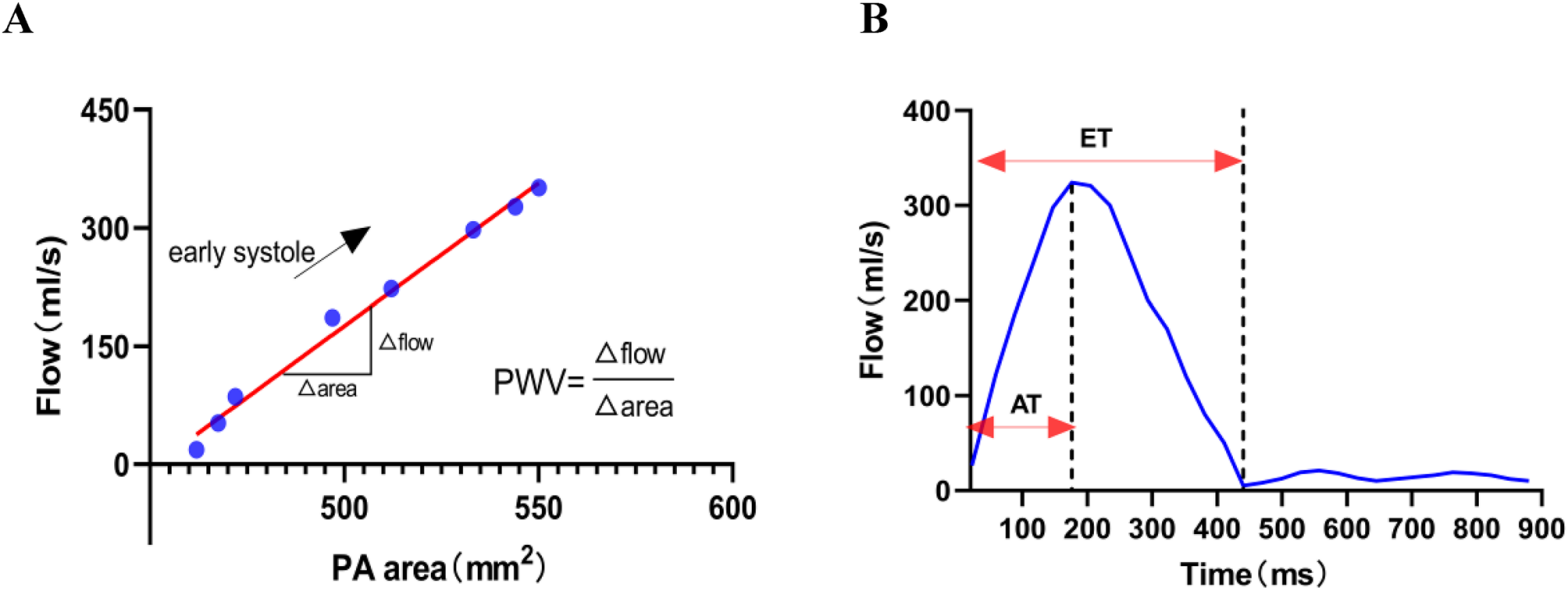
Method for PA PWV, AT and ET measurements. (**A**) The plot shows the flow area of the PA of eight cardiac phases during early systole. The slope of a fitted line to the early systole gives Δflow/Δarea = PWV. (**B**) The plot shows the time course of the PA blood flow. The left dotted vertical line is the time of peak systolic flow. The right dotted vertical line is the time of the end of ejection. AT is the time interval between the onset of the PA blood flow and the peak flow (short red double arrow). ET is the time interval between the onset and the end of systolic PA blood flow (long red double arrow). *PA* pulmonary artery, *AT* acceleration time, *ET* ejection time.

CMR images were analyzed offline at our CMR-core lab by a single observer, who was blinded to the clinical data, using specialized software (Medis Suite, version 3.1, Leiden, The Netherlands). LV contours were outlined manually on the cardiac cine images using QMass 8.1. The contours of the PA were semiautomatically traced and, where needed, manually adjusted for each phase using QFlow 8.1. The PA maximum cross-sectional area (A_max_) and minimum area (A_min_) were acquired from the end-systolic and end-diastolic (either magnitude or phase) images, respectively (Figs. 1, 2).

PA stiffness indices were assessed in two categories: (a) compliance and (b) flow velocities, primarily using PA relative area change (RAC) and pulse wave velocity (PWV), both of which have been shown to predict outcomes in patients with pre-capillary PH^10,11,24^. The main compliance parameter analyzed was the RAC based on the PA area change (AC). The AC was defined as AC = A_max_ − A_min_, RAC was defined respectively as RAC = (A_max_ − A_min_)/A_min_. A smaller area change (both AC and RAC) indicates a stiffer vessel.

PWV was evaluated as the ratio between flow variation (Δflow) and area variation (Δarea) by the flow area (QA) method^24–26^. The PWV values were derived from the slope of a line fitted to the early systole (Fig. 2). Other hemodynamic parameters assessed included acceleration time (AT), ejection time (ET), flow per min (FPM), mean pressure gradient (MPG), net flow volume (NFV), peak flow velocity (PV), and peak pressure gradient (PPG).

AT and ET were determined as the time interval between the onset of blood flow and peak flow and the time interval between the onset and end of systolic blood flow, respectively (Fig. 2).

To assess the intra- and interobserver reproducibility, 10 random cases (5 healthy volunteers and 5 HF patients) were analyzed by 2 observers (X.H and P.B.) blinded to the clinical data at 2 separate times (median 5 days apart from eachother).

### Statistical analysis

Data for categorical variables are expressed as percentages and were compared by chi-square test. Continuous data were tested for normality with the Shapiro–Wilk test. Normally distributed continuous variables are presented as mean ± standard deviation, and differences between groups were compared using independent t-tests or analysis of variance. Non-normally distributed continuous variables are presented as medians (interquartile ranges), and different groups were compared with the nonparametric Mann–Whitney U test or the Kruskal–Wallis test as appropriate. Multivariate linear regression was performed to adjust for age differences in baseline characteristics. Correlations between variables were explored using the Pearson or Spearman correlation coefficients, and further evaluation of relationships between parameters was performed with linear regression analysis. Intra- and interobserver variability were tested by Bland–Altman agreement analysis, intraclass correlation coefficient, and coefficient of variation. The analyses were performed with dedicated statistical packages (SPSS 25.0, Chicago, USA; GraphPad Prism 8.0, San Diego, USA; MedCalc 19.0, Belgium).

### Ethics approval and consent to participate

As described above, the study complied with the Declaration of Helsinki, the protocols were approved by the responsible ethics committees, and all patients gave written informed consent. The study was registered at the German Clinical Trials Register (DRKS, registration number: DRKS00015615).

## Results

### Population characteristics

A total of 74 subjects were prospectively enrolled in this study: control subjects (n = 19) and HF patients (n = 55), who were further divided into 3 HF subgroups: HFpEF group (n = 20), HFmrEF group (n = 18), and HFrEF group (n = 17). In line with previously published analyses, baseline characteristics are presented in Table 1 for completeness^19–23^.

**Table 1:** Baseline characteristics of the study cohort.

Variable	Controls (n = 19)	HFpEF (n = 20)	HFmrEF (n = 18)	HFrEF (n = 17)	p value		
					Controls vs. HFpEF	Controls vs. HFmrEF	Controls vs. HFrEF
Age (years)	62 ± 9	76 ± 9	67 ± 10	65 ± 11	< 0.001	0.059	0.718
Men	9 (53%)	11 (55%)	12 (67%)	14 (82%)	0.376	0.155	0.022
BMI (kg/m^2^)	25.48 ± 3.28	27.60 ± 3.55	26.35 ± 4.19	28.99 ± 3.78	0.218	0.402	0.104
Smoking	0 (0)	9 (45%)	14 (78%)	11 (65%)	0.001	< 0.001	< 0.001
Arterial hypertension	0 (0)	16 (80%)	15 (83%)	13 (76%)	< 0.001	< 0.001	< 0.001
Hypercholesterolemia	0 (0)	12 (60%)	13 (72%)	10 (59%)	< 0.001	< 0.001	< 0.001
Diabetes mellitus	0 (0)	5 (25%)	3 (17%)	5 (29%)	0.020	0.063	0.011
Stroke	0 (0)	2 (10%)	0 (0)	1 (6%)	0.157	–	0.284
COPD	0 (0)	1 (5%)	3 (17%)	0 (0)	0.323	0.063	–
CAD	0 (0)	11 (55%)	15 (83%)	13(76%)	< 0.001	< 0.001	< 0.001
Previous MI	0 (0)	1 (5%)	3 (17%)	5 (29%)	0.323	0.063	0.011
NYHA functional class II	0 (0)	12 (60%)	14 (78%)	12 (71%)	< 0.001	< 0.001	< 0.001
NYHA functional class III	0 (0)	8 (40%)	4 (22%)	5 (29%)	0.002	0.030	0.011
6 MWD (m)	522.9 ± 118.6	358.4 ± 84.0	400.8 ± 12.6	432.4 ± 85.6	0.001	0.007	0.019
Brachial artery hemodynamics							
Systolic BP (mmHg)	128 ± 10	128 ± 20	122 ± 17	117 ± 17	0.866	0.421	0.094
Diastolic BP (mmHg)	72 ± 6	68 ± 11	69 ± 9	68 ± 9	0.237	0.370	0.293
Pulse pressure (mmHg)	56 ± 9	60 ± 15	54 ± 12	49 ± 12	0.280	0.647	0.122
Cardiac MRI							
LVEF (%)	64 ± 5	61 ± 4	45 ± 3	33 ± 5	0.034	< 0.001	< 0.001
Blood explorations							
CRP (mg/L)	1.2 ± 1.4	2.9 ± 2.5	2.9 ± 4.1	1.2 ± 0.7	0.072	0.051	0.838
NT-proBNP (ng/L)	88 ± 61	529 ± 601	829 ± 1158	2247 ± 3447	< 0.001	< 0.001	< 0.001
Medication							
Diuretic	0 (0)	8 (40%)	9 (50%)	6 (35%)	0.002	< 0.001	0.005
ACE inhibitors	0 (0)	6 (30%)	7 (39%)	8 (47%)	0.004	0.003	0.001
AR blocker	0 (0)	10 (50%)	10 (56%)	8 (47%)	< 0.001	< 0.001	0.001
Calcium channel blockers	0 (0)	3 (15%)	2 (11%)	1 (6%)	0.079	0.135	0.284
Beta-Blockers	0 (0)	10 (50%)	12 (67%)	15 (88%)	< 0.001	< 0.001	< 0.001

Data are expressed as the mean ± standard deviation or n (%).

*HV* healthy volunteer, *HFpEF* heart failure with preserved ejection fraction, *HFmrEF* heart failure with mid-range ejection fraction, *HFrEF* heart failure with reduced ejection fraction, *BMI* body mass index, *LVEF* left ventricular ejection fraction, *COPD* chronic obstructive pulmonary disease, *CAD* coronary artery disease, *MI* myocardial infarction, *NYHA* New York Heart Association, *6MWD* 6 min walk distance, *BP* blood pressure, *E* early diastolic peak (pulsed-wave Doppler), *e'* early diastolic mitral annular velocity by Doppler tissue imaging, *sPAP* systolic pulmonary artery pressure, *CRP* C-reactive protein, *NT-proBNP* N-terminal pro brain natriuretic peptide, *ACE* angiotensin converting enzyme, *AR* angiotensin receptor.

### PA stiffness and flow parameters

**Figure 3:**
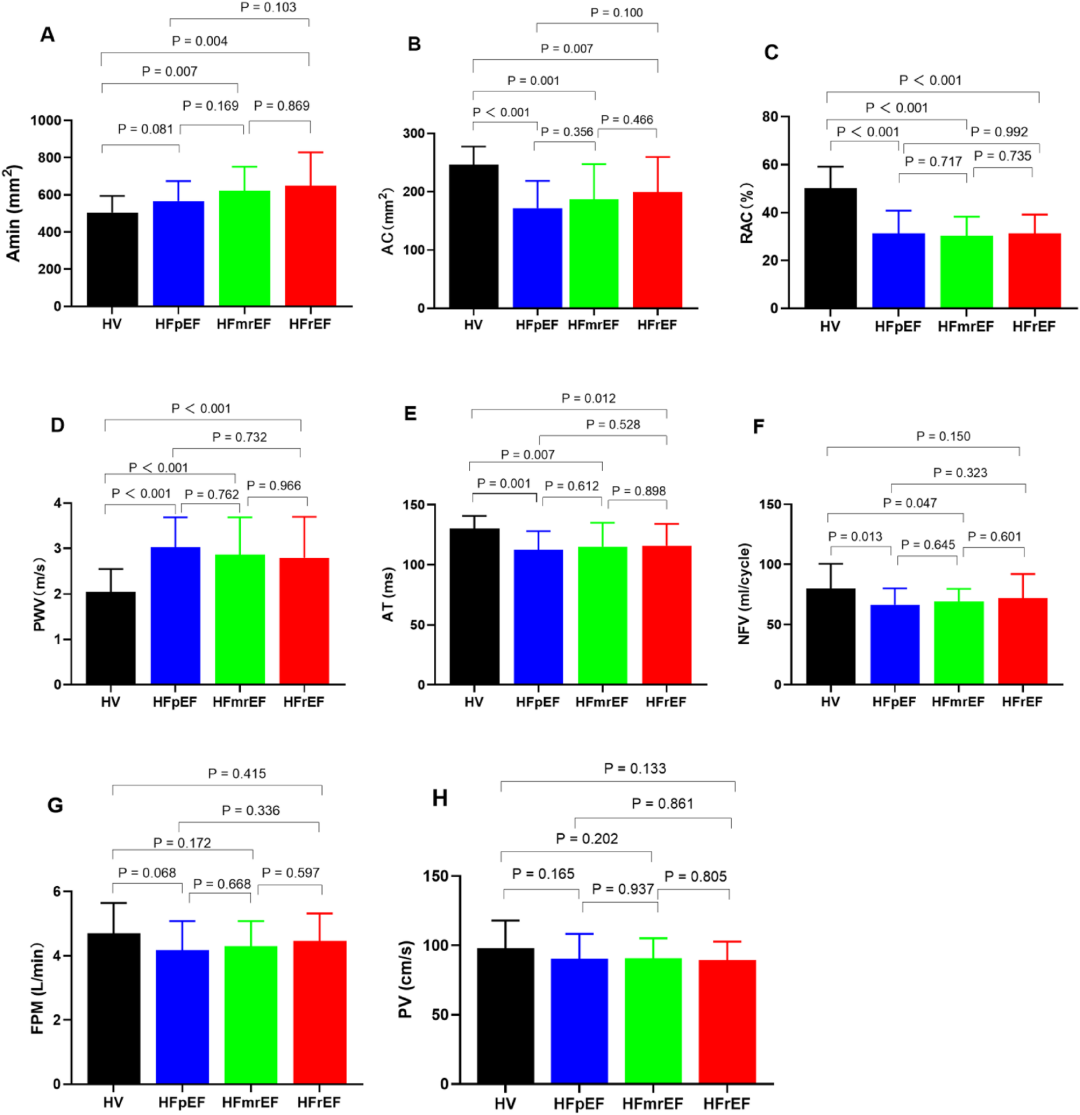
Comparison of PA stiffness and flow hemodynamics across all groups. Graphs with mean values and standard deviations for pulmonary artery A_min_ (**A**), AC (**B**), RAC (**C**), PWV (**D**), AT (**E**), NFV (**F**), FPM (**G**), and PV (**H**) in the controls group and HF subgroups. *A*_*min*_ minimum pulmonary artery across-sectional area, *AC* area change, *AT* acceleration time, *FPM* flow per min, *HFpEF* heart failure with preserved ejection fraction, *HFmrEF* heart failure with mid-range ejection fraction, *HFrEF* heart failure with reduced ejection fraction heart failure, *HV* healthy volunteer, i.e. control subject, *NFV* net flow volume, *PV* peak flow velocity, *PWV* pulse wave velocity, *RAC* relative area change.

**Figure 4:**
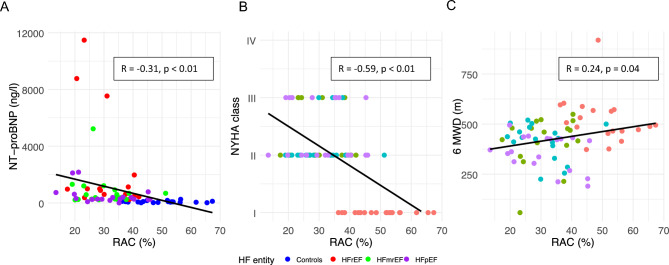
Correlation between NT-proBNP level or functional capacity (NYHA class and 6MWD) and PA RAC. The graphs show the results of the linear regression analysis in the entire study between NT-proBNP and RAC (**A**), NYHA class and RAC (**B**), and 6 MWD and RAC (**C**). Green solid circles represent the control group, whereas purple squares, light blue triangles and red triangles represent the HFpEF group, HFmrEF group and HFrEF group, respectively. Black solid lines are the fitted lines of regression analysis for all datasets. *6MWD* 6 min walk distance, *NYHA class* New York Heart Association functional class, *HFpEF* heart failure with preserved ejection fraction, *HFmrEF* heart failure with mid-range ejection fraction, *HFrEF* heart failure with reduced ejection fraction heart failure, *HV* healthy volunteer, i.e. control subject, *NT-proBNP* N-terminal pro brain natriuretic peptide, *RAC* relative area change.

In in the pooled HF group as well as in all three HF groups separately, PA proved to be stiffer than in the control group (Tables 2, 3, Figs. 3, 4). All HF patient groups showed an increased PA stiffness with reduced RAC and increased PWV as compared to control subjects.

**Table 2:** CMR data in healthy subjects and all HF patients.

Variable	Total (n = 74)	Controls (n = 19)	HF (n = 55)	p value
A_max_ (mm^2^)	770.23 (660.03–852.30)	779.69 (656.79–824.32)	756.09 (660.53–902.36)	0.524
A_min_ (mm^2^)	563.83 (484.77–650.48)	537.40 (423.93–593.00)	584.36 (510.76–662.40)	0.003
AC (mm^2^)	201.13 ± 57.12	246.61 ± 31.09	185.42 ± 5575	< 0.001
RAC (%)	35.84 ± 12.02	50.08 ± 9.08	30.92 ± 8.47	< 0.001
PWV (m/s)	2.80 ± 0.66	2.11 ± 0.48	3.03 ± 0.53	< 0.001
AT (ms)	118 ± 18	130 ± 11	114 ± 18	0.001
ET (ms)	337 ± 35	349 ± 33	332 ± 36	0.069

Data are represented as the mean ± standard deviation or median (interquartile range).

*A*_*max*_ maximum pulmonary artery cross-sectional area, *A*_*min*_ minimum pulmonary artery across-sectional area, *AC* area change, *AT* acceleration time, *ET* ejection time, *PWV* pulse wave velocity, *RAC* relative area change.

**Table 3:** CMR-derived PA stiffness and flow hemodynamics in healthy subjects and HF subgroups.

Variable	Controls (n = 19)	HFpEF (n = 20)	HFmrEF (n = 18)	HFrEF (n = 17)	Pairwise comparison p value		
					Controls vs. HFpEF	Controls vs. HFmrEF	Controls vs. HFrEF
A_max_ (mm^2^)	779.69 (656.79–824.32)	726.54 (639.74–804.34)	794.29 (660.03–981.91)	814.50 (674.79–978.21)	0.482	0.288	0.188
A_min_ (mm^2^)	537.40 (423.93–593.00)	562.16 (483.25–622.27)	629.34 (536.11–698.56)	610.11 (535.10–703.61)	0.081	0.007	0.004
AC (mm^2^)	246.61 ± 31.09	171.85 ± 46.65	187.09 ± 60.28	199.60 ± 59.99	< 0.001	0.001	0.007
RAC (%)	50.08 ± 9.08	31.26 ± 9.57	30.22 ± 8.08	31.23 ± 7.95	< 0.001	< 0.001	< 0.001
PWV (m/s)	2.11 ± 0.48	3.07 ± 0.56	3.02 ± 0.49	3.01 ± 0.56	< 0.001	< 0.001	< 0.001
AT (ms)	130 ± 11	112 ± 16	115 ± 20	115 ± 18	0.001	0.007	0.012
ET (ms)	349 ± 33	329 ± 39	333 ± 36	334 ± 32	0.085	0.176	0.196

Data are represented as the mean ± standard deviation or median (interquartile range).

*A*_*max*_ maximum pulmonary artery cross-sectional area, *A*_*min*_ minimum pulmonary artery across-sectional area, *AC* area change, *AT* acceleration time, *ET* ejection time, *FPM* flow per min, *MPG* mean pressure gradient, *PWV* pulse wave velocity, *RAC* relative area change.

The remaining hemodynamic parameters assessed, including NFV, FPM, PV, PPG, and MPG, were also altered in the HF groups compared to the controls (S2 and S3). The measurements of both PA stiffness and flow parameters are consistent, e.g. the higher the PA stiffness was, the higher was the pulse wave velocity. A summary of the correlations is given in the supplementary information (S4).

### Comparison between groups

All HF groups showed an increase in PA stiffness compared to control subjects (Table 3), e.g. RAC was highest in controls, while the HF group RAC values were similar (Table 3).

### Correlation with symptom burden

Both clinical and laboratory measures of disease severity were associated with PA stiffness and altered flow parameters. Patients with a higher clinical symptom burden, assessed by both NYHA functional class and 6MWD, had stiffer PA parameters and altered flow parameters (Fig. 4, Fig. S6). This association was also confirmed by a laboratory surrogate of disease burden (NT-proBNP), i.e. a lower RAC was associated with a higher NT-proBNP level (r = − 0.31, p < 0.01) and a higher NYHA class (r = − 0.59, p ≤ 0.01).

### Reproducibility of measurements

Intra- and interobserver variability was low for all CMR measurements, supporting a high reliability of the measurements (S6 and S7).

## Discussion

In this study, we quantitatively assessed for the first time PA stiffness and blood flow dynamics using advanced CMR in HF patient including all three HF entities and in healthy subjects without HF. We demonstrate that (a) PA stiffness is higher in all three HF groups compared to control subjects and PA hemodynamic parameters are consistently altered. Furthermore, we (b) detected significant correlations between the presence and degree of PA stiffness and the disease burden with regards to both clinical and laboratory HF parameters. Comparisons between the different HF entities revealed (c) that PA stiffness is similar between the three HF entities.

Acknowledging the similarity across HF entities indicates that PA stiffness may be independent from the systemic vascular changes due to the RAAS system and RAAS inhibiting therapeutic effects.

PA wall stiffness is considered an important determinant of cardiovascular risk at an early stage of pulmonary disease^11,27^. Changes in vascular mechanics often precede gross remodeling; thus, an assessment of PA stiffness can be a useful tool in the early identification of pathological changes in the pulmonary vasculature—which has already been proven in PAH, but never in PH in the context of HF^10,11,24,28,29^.

We detected important differences in the indices of PA stiffness between control group and HF patients. There was a significant reduction in PA absolute AC, RAC and AT, as well as a significant elevation in PA PWV in patients with HF. The differences in PA stiffness measurements observed in our study are consistent with findings from previous studies that employed both CMR and right heart catheterization as diagnostic tools. Specifically, 2 studies primarily focused on PA stiffness and its predictive value in pulmonary arterial hypertension^9,11^. Three complementary studies concentrated more on right heart/pulmonary artery pressures and their associated CMR-determined RV changes^16–18^. Therefore, our results add to the growing body of literature supporting the clinical relevance of PA stiffness measurements. A previous report demonstrated that PH in patients with HF is common^30^. Consistently, we showed for the first time mild pulmonary hypertension in HF patients by CMR. In the HF group, especially in the HFmrEF and HFrEF subgroups, we found significant PA lumen dilatation (A_min_, Table 3), which suggests long-term volume overload due to left-sided heart disease and/or abnormal changes in pulmonary vessel walls due to pathological factors.

Recent data show that abnormal PA hemodynamic status occurs in the early stages of HF and is strongly associated with unfavorable outcomes in HF^5,6,31^. Prior studies showed that PA flow hemodynamic parameters were significantly decreased in PH and chronic thromboembolic pulmonary hypertension (CTEPH) compared to healthy subjects^11,16,17,32^. Similar alterations in PA flow hemodynamics were detected for NFV, FPM, and PV in our study.

Previous studies have shown that increasing PA stiffness is associated with worsening functional parameters in patients with PH and in those with HF^13,33,34^.

Our measurements appear to be reliably assessed as indicated by our intra-observer and inter-observer variability assessment with a high reproducibility of measurements. These results are in line with previous studies that tested observer variability for PA imaging by CMR in other cohorts^33^.

Our observation requires a prospective, follow-up analysis or a confirmation in a cohort followed longitudinally to assess the prognostic relevance of PA stiffness and PA hemodynamics. PA stiffness may present a central characteristic of HF patients with the potential to serve as a relevant parameter to better characterize and group HF patients.

### Limitations

This study was a single-center study. Thus, a center-specific bias could not be excluded. However, single-center data collection bears several advantages: inclusion of a homogenous patient population; a standard work-up routine; and consistent quality of the CMR examination. In addition, there was a small sample size of the various HF subgroups. Therefore, it is difficult to extrapolate these results to a general population. Although we divided our subjects into HF subgroups according to the most recent guidelines, HF is a complex clinical syndrome with various etiologies that could impact the changes in PA hemodynamics. The absence of long-term follow-up data for this patient cohort restricts our ability to evaluate the long-term clinical significance of our findings.

## Conclusions

CMR is a reproducible noninvasive technique to assess PA stiffness and flow hemodynamics. As we could demonstrate for the first time in the context of HF patients an post-capillary PH, CMR is able to detect significant differences in stiffness and blood flow hemodynamics between subjects without HF and HF patients. HF patients show higher values for PA stiffness compared to controls subjects.

### Supplementary Information

Supplementary Information.

## Data Availability

The data can be accessed upon request with a research proposal submitted to the investigator via the corresponding author.

## Abbreviations

AC: Area change
Amax: Maximum pulmonary artery cross-sectional area
Amin: Minimum pulmonary artery across-sectional area
AT: Acceleration time
CMR: Cardiovascular magnetic resonance imaging
CTEPH: Chronic thromboembolic pulmonary hypertension
CV: Cardiovascular
ET: Ejection time
FPM: Flow per min
HF: Heart failure
HFmrEF: Heart failure with preserved ejection fraction
HFpEF: Heart failure with mildly-reduced ejection fraction
HFrEF: Heart failure with reduced ejection fraction
LVEF: Left ventricular ejection fraction
MPG: Mean pressure gradient
NFV: Net flow volume
PA: Pulmonary artery
PAH: Pulmonary arterial hypertension
PCMR: Phase contrast magnetic resonance
PPG: Peak pressure gradient
PV: Peak flow velocity
PWV: Pulse wave velocity
RAC: Relative area change
