# Supplementary material for: Noninvasive evaluation of pulmonary artery stiffness in heart failure patients via cardiovascular magnetic resonance
Source: Sci Rep. 2023 Dec 19;13:22656. doi: 10.1038/s41598-023-49325-5 (PMC10730605; doi:10.1038/s41598-023-49325-5)
Supplement: Supplementary file 1 — Supplementary Information. [file 41598_2023_49325_MOESM1_ESM.docx]

**SUPPLEMENTARY INFORMATION**

**S1 Example of a cross-sectional image of the PA using phase-contrast through-plane sequences**

**S2 CMR data in healthy subjects and all HF patients**

**S3 CMR-derived PA stiffness and flow hemodynamics in healthy subjects and HF subgroups**

**S4 Correlations between CMR derived PA parameters**

**S5 Correlation between clinical data and CMR-derived PA parameters**

**S6 Bland–Altman Analyses of the Intra- and Interobserver Variability of CMR Imaging Measurements**

**S7 Reproducibility of CMR measurements**

**S1 – Example of a cross-sectional image of the PA using phase-contrast through-plane sequences**

**
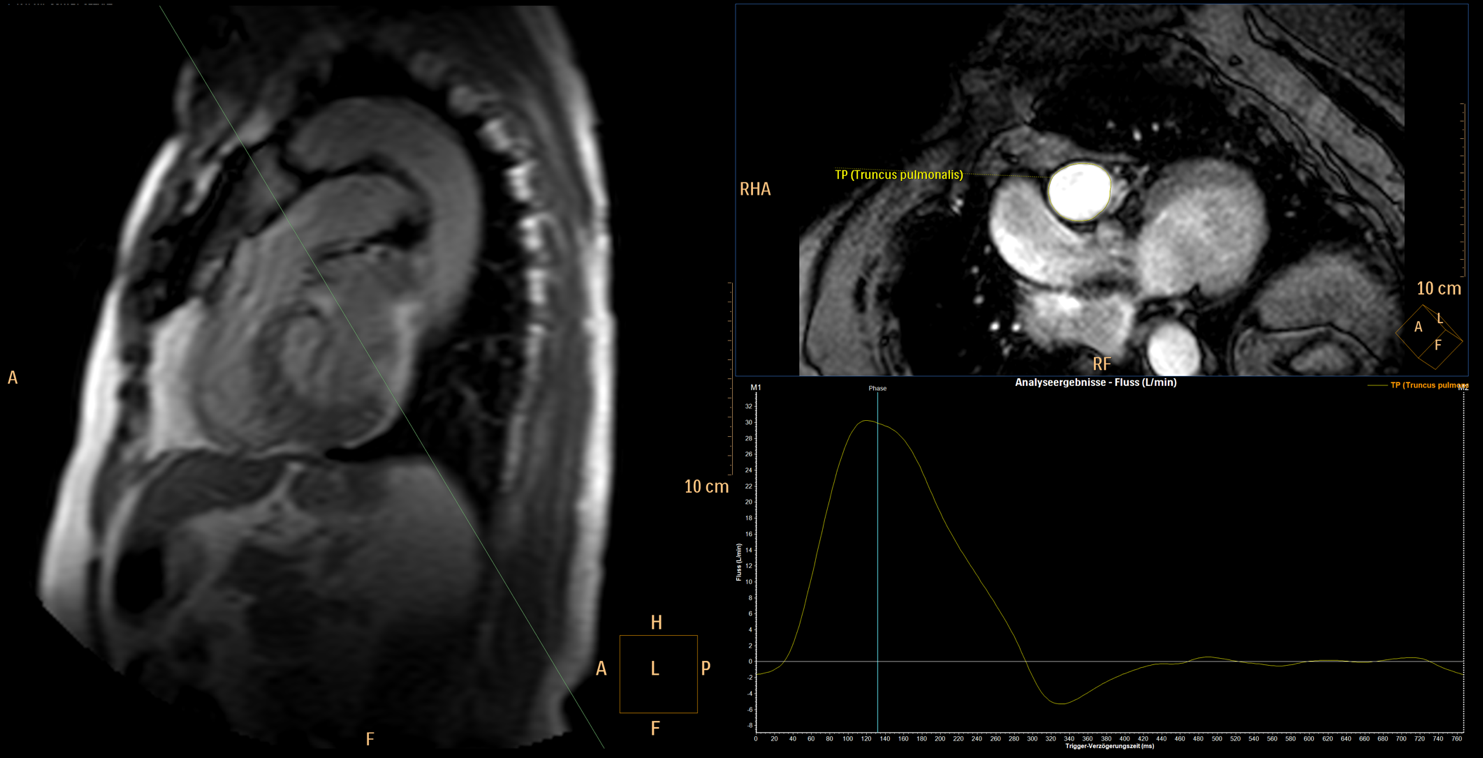
**

**S2 – CMR data in healthy subjects and all HF patients**

| **Variable** | **Total** | **Controls** | **HF** | **P Value** |
| --- | --- | --- | --- | --- |
| Number of subjects | 74 | 19 | 55 |  |
| A_max_ (mm^2^) | 770.23 (660.03-852.30) | 779.69 (656.79-824.32) | 756.09 (660.53-902.36) | 0.524 |
| A_min_ (mm^2^) | 563.83 (484.77-650.48) | 537.40 (423.93-593.00) | 584.36 (510.76-662.40) | 0.003 |
| AC (mm^2^) | 201.13 ± 57.12 | 246.61 ± 31.09 | 185.42 ± 5575 | < 0.001 |
| RAC (%) | 35.84 ± 12.02 | 50.08 ± 9.08 | 30.92 ± 8.47 | < 0.001 |
| PWV (m/sec) | 2.80 ± 0.66 | 2.11 ± 0.48 | 3.03 ± 0.53 | < 0.001 |
| AT (ms) | 118 ± 18 | 130 ± 11 | 114 ± 18 | 0.001 |
| ET (ms) | 337 ± 35 | 349 ± 33 | 332 ± 36 | 0.069 |
| NFV (ml/cycle) | 71.96 ± 17.05 | 80.13 ± 20.30 | 69.13 ± 14.96 | 0.014 |
| FPM (L/min) | 4.40 ± 0.88 | 4.70 ± 0.94 | 4.30 ± 0.84 | 0.093 |
| PPG (mmHg) | 3.62 ± 1.31 | 4.00 ± 1.70 | 3.49 ± 1.13 | 0.144 |
| MPG (mmHg) | 1.12 ± 0.33 | 1.20 ± 0.26 | 1.09 ± 0.35 | 0.182 |
| PV (cm/s) | 92.21 ± 16.72 | 97.91 ± 19.97 | 90.24 ± 15.15 | 0.085 |

Data are represented as the mean ± standard deviation or median (interquartile range).

A_max_ = maximum pulmonary artery cross-sectional area；A_min_ = minimum pulmonary artery across-sectional area; AC = area change; AT = acceleration time; ET = ejection time; FPM = flow per min; MPG = mean pressure gradient; NFV = net flow volume; PPG = peak pressure gradient; PV = peak flow velocity; PWV = pulse wave velocity; RAC = relative area change.

**S3 – CMR-derived PA stiffness and flow hemodynamics in healthy subjects and HF subgroups**

| **Variable** | **Controls**  **(n = 19)** | **HFpEF**  **(n = 20)** | **HFmrEF**  **(n = 18)** | **HFrEF**  **(n = 17)** | **P Value** | | |
| --- | --- | --- | --- | --- | --- | --- | --- |
|  |  |  |  |  | **Controls**  **vs. HFpEF** | **Controls**  **vs. HFmrEF** | **Controls**  **vs. HFrEF** |
| NFV (ml/cycle) | 80.13 ± 20.30 | 66.63 ± 13.54 | 69.13 ± 10.73 | 72.07 ± 19.98 | 0.013 | 0.047 | 0.150 |
| FPM (L/min) | 4.70 ± 0.94 | 4.18 ± 0.91 | 4.29 ± 0.78 | 4.45 ± 0.85 | 0.068 | 0.172 | 0.415 |
| PPG (mmHg) | 4.00 ± 1.70 | 3.55 ± 1.40 | 3.50 ± 0.85 | 3.40 ± 1.11 | 0.294 | 0.249 | 0.181 |
| MPG (mmHg) | 1.20 ± 0.26 | 1.07 ± 0.35 | 1.08 ± 0.40 | 1.10 ± 0.29 | 0.221 | 0.264 | 0.394 |
| PV (cm/s) | 97.91 ± 19.97 | 90.40 ± 17.85 | 90.83 ± 14.35 | 89.43 ± 13.27 | 0.165 | 0.202 | 0.133 |

Data are represented as the mean ± standard deviation or median (interquartile range).

A_max_ = maximum pulmonary artery cross-sectional area；A_min_ = minimum pulmonary artery across-sectional area; AC = area change; AT = acceleration time; ET = ejection time; FPM = flow per min; MPG = mean pressure gradient; NFV = net flow volume; PPG = peak pressure gradient; PV = peak flow velocity; PWV = pulse wave velocity; RAC = relative area change.

**S4 – Correlations between CMR derived PA parameters**

**
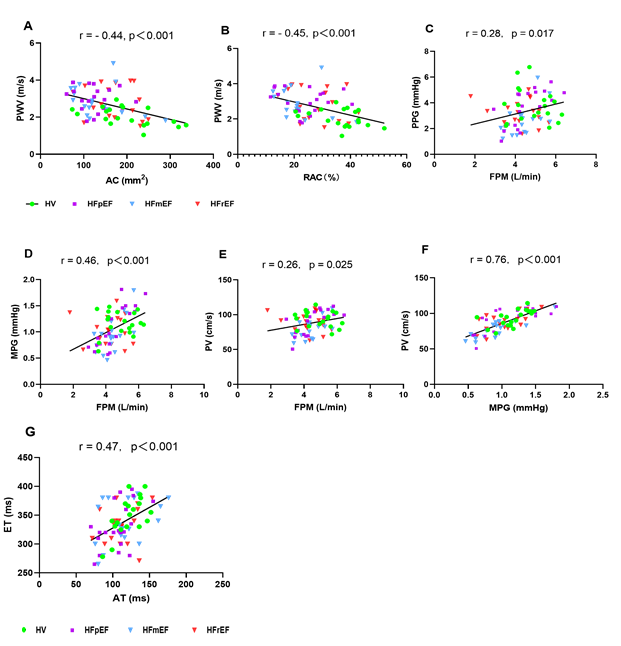
**

The graphs show the results of correlation analysis in the whole study between PWV and AC (A), PWV and RAC (B), PPG and FPM (C), MPG and FPM (D), PV and FPM (E), PV and MPG (F), and AT and ET (G). PPG = peak pressure gradient; MPG = mean pressure gradient; AT = acceleration time; ET = ejection time; for other abbreviations see Figure 3. Green solid circles represent the control group, whereas purple squares, light blue triangles and red triangles represent the HFpEF group, HFmrEF group and HFrEF group, respectively. Black solid lines are the fitted lines of the regression analysis for all datasets.

**S5** – **Correlation between clinical data and CMR-derived PA parameters**

| **Variable** | **NT-proBNP** | | **NYHA Class** | | **6 MWD** | |
| --- | --- | --- | --- | --- | --- | --- |
|  | **r** | **P value** | **r** | **P value** | **r** | **P value** |
| Age | 0.18 | 0.120 | 0.41 | <0.001 | - 0.50 | <0.001 |
| BMI | - 0.07 | 0.533 | 0.26 | 0.024 | - 0.16 | 0.156 |
| LVEF | - 0.44 | 0.001 | -0.43 | 0.001 | 0.08 | 0.611 |
| A_max_ | 0.19 | 0.092 | 0.08 | 0.495 | - 0.04 | 0.691 |
| A_min_ | 0.30 | 0.009 | 0.28 | 0.013 | - 0.12 | 0.307 |
| PWV | 0.25 | 0.036 | 0.71 | <0.001 | - 0.43 | <0.001 |
| AC | - 0.18 | 0.129 | - 0.47 | <0.001 | 0.16 | 0.173 |
| RAC | - 0.31 | 0.007 | - 0.66 | <0.001 | 0.24 | 0.039 |
| NFV | - 0.04 | 0.734 | - 0.41 | <0.001 | 0.34 | 0.003 |
| FPM | - 0.08 | 0.493 | - 0.33 | 0.003 | 0.29 | 0.010 |
| PPG | - 0.14 | 0.220 | - 0.21 | 0.061 | 0.05 | 0.618 |
| MPG | - 0.13 | 0.261 | - 0.22 | 0.054 | 0.16 | 0.164 |
| PV | - 0.22 | 0.062 | - 0.26 | 0.027 | 0.13 | 0.264 |
| AT | - 0.27 | 0.020 | - 0.35 | 0.002 | 0.26 | 0.024 |
| ET | - 0.10 | 0.360 | - 0.21 | 0.068 | 0.02 | 0.867 |

Data are represented as the mean ± standard deviation or median (interquartile range).

A_max_ = maximum pulmonary artery cross-sectional area；A_min_ = minimum pulmonary artery across-sectional area; AC = area change; AT = acceleration time; ET = ejection time; FPM = flow per min; MPG = mean pressure gradient; NFV = net flow volume; PPG = peak pressure gradient; PV = peak flow velocity; PWV = pulse wave velocity; RAC = relative area change.

**S6 – Bland–Altman Analyses of the Intra- and Interobserver Variability of CMR Imaging Measurements**

**
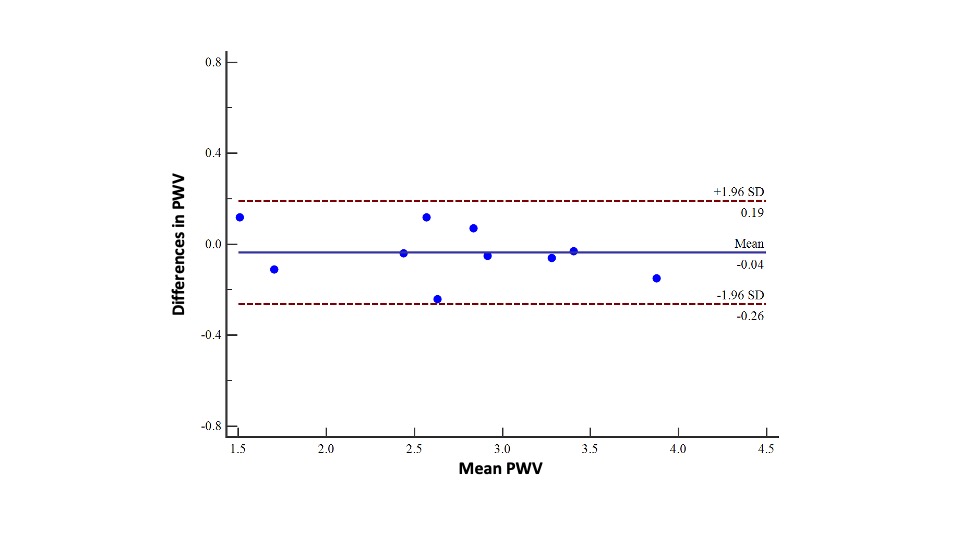
A1 A2**

**
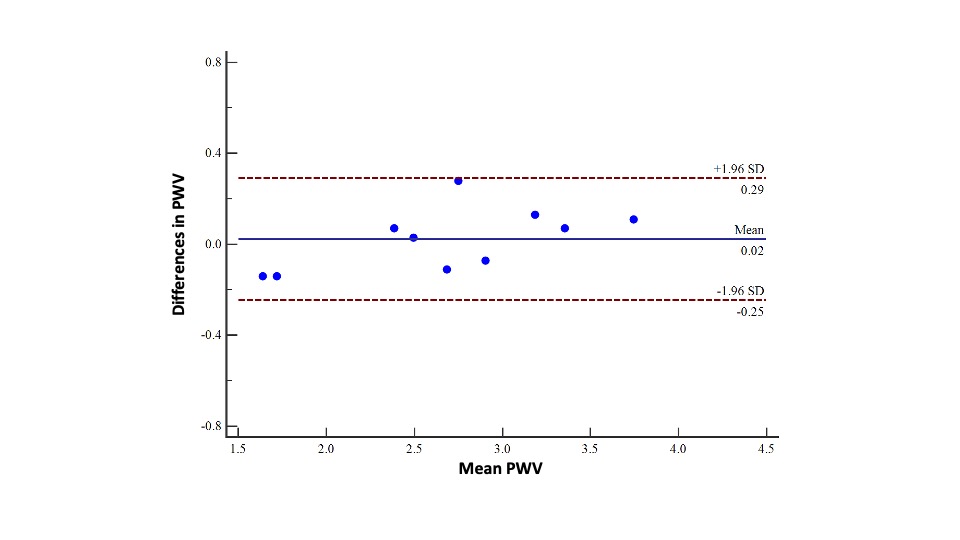
**

**
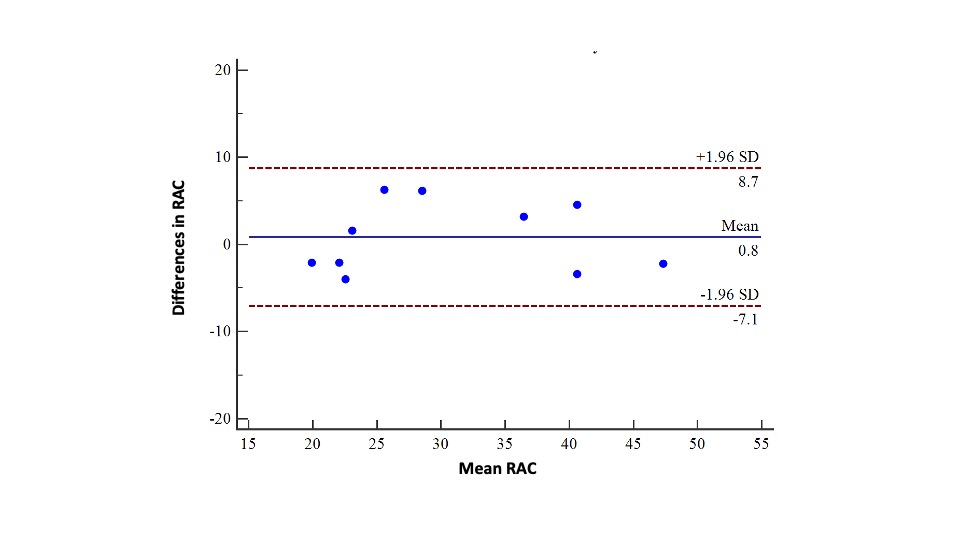

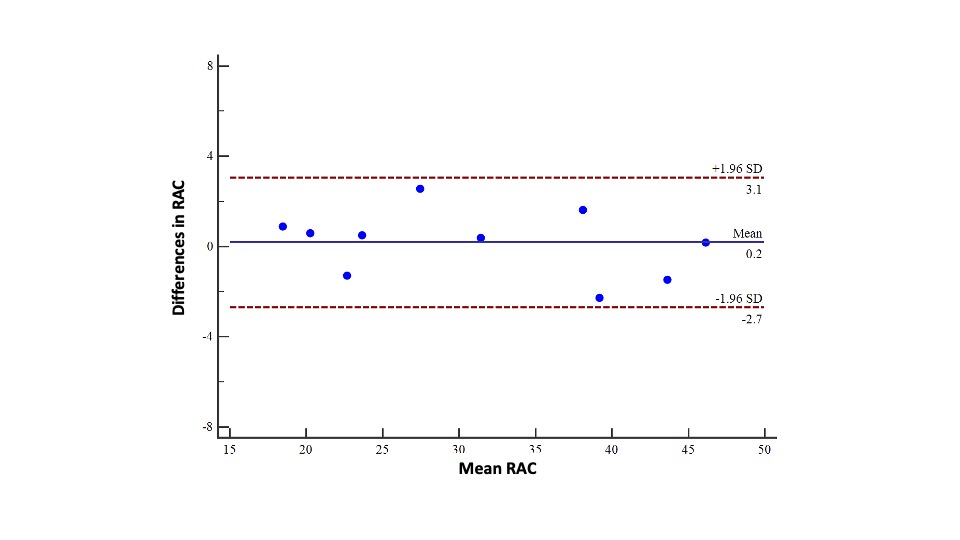
B1 B2**

**
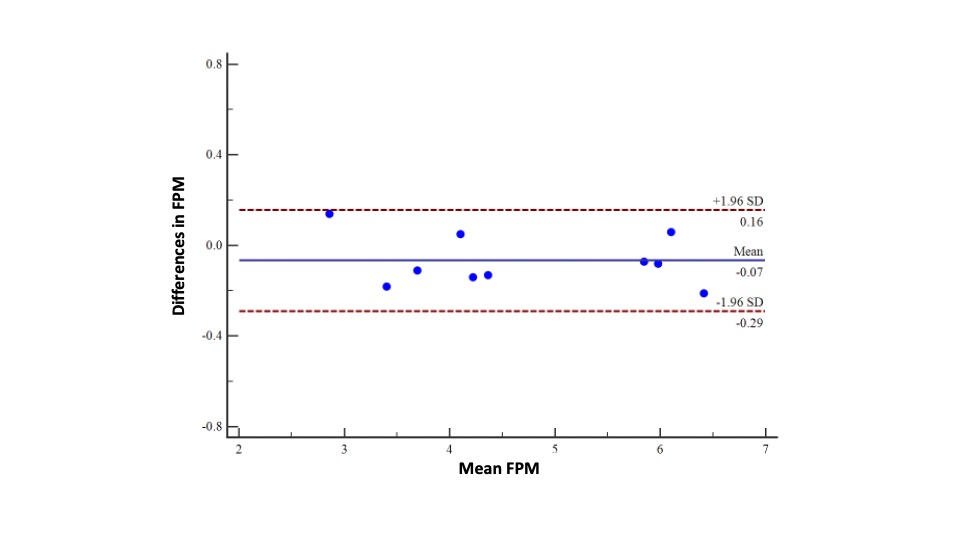

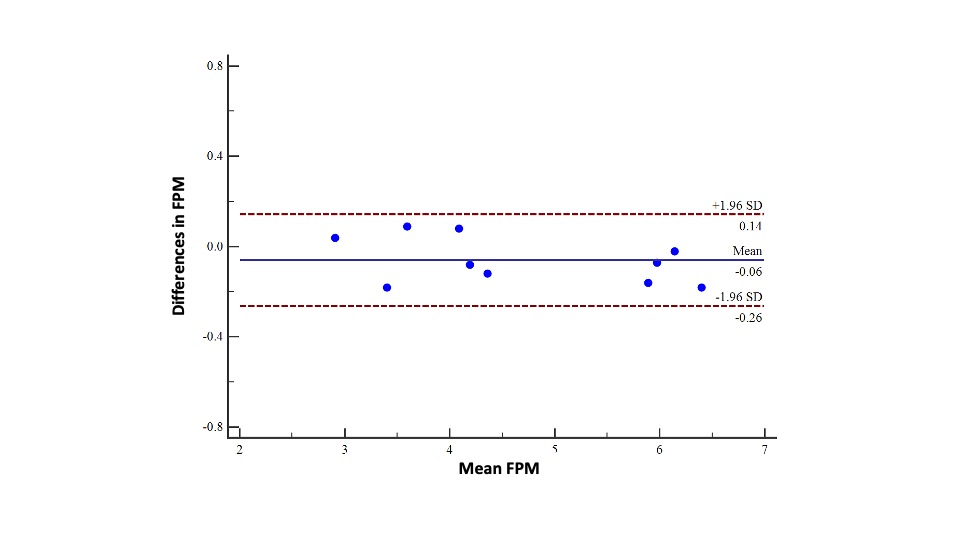
C1 C2**

Bland–Altman plots demonstrating the intraobserver (1) and interobserver (2) agreement of measurements for PWV (A), RAC (B), and FPM (C). The middle-solid line is the mean difference of the measures. The upper and lower dashed lines are ±1.96 standard deviation.

**S7 – Reproducibility of CMR measurements**

|  | **Bland–Altman*** | | **ICC** | | **CoV (**%**)** | |
| --- | --- | --- | --- | --- | --- | --- |
|  | **Intraobserver** | **Interobserver** | **Intra-**  **observer** | **Inter-**  **observer** | **Intra-**  **observer** | **Inter-**  **observer** |
| Amax | 0.7 (-13.9 to 15.3) | - 2.1 (-24.2 to 20.0) | 0.99 | 0.98 | 2.6 | 3.4 |
| Amin | - 0.6 (-14.8 to 13.6) | - 3.1 (-19.9 to 13.8) | 0.99 | 0.98 | 3.1 | 3.6 |
| PWV | - 0.04 (-0.26 to 0.19) | 0.02 (-0.25 to 0.29) | 0.98 | 0.97 | 4.5 | 5.3 |
| AC | - 0.3 (-14.1 to 13.5) | - 0.8 (-16.3 to 14.7) | 0.98 | 0.97 | 4.9 | 5.8 |
| RAC | 0.2 (-2.7 to 3.1) | 0.8 (-7.1 to 8.7) | 0.97 | 0.96 | 5.0 | 6.2 |
| NFV | 0.6 (-11.0 to 12.2) | 1.0 (-13.6 to 15.6) | 0.98 | 0.97 | 4.8 | 5.9 |
| FPM | - 0.06 (-0.26 to 0.14) | - 0.07 (-0.29 to 0.16) | 0.99 | 0.98 | 3.3 | 3.9 |
| AT | - 1.7 (-13.2 to 9.8) | - 1.8 (-20.3 to 16.7) | 0.97 | 0.95 | 5.9 | 7.5 |
| ET | - 7.6 (-24.3 to 9.1) | - 8.9 (-28.4 to 10.6) | 0.96 | 0.94 | 7.2 | 8.1 |
| PV | - 1.1 (-12.8 to 10.7) | 1.5 (-17.3 to 20.3) | 0.97 | 0.95 | 5.6 | 7.6 |
| PPG | - 0.04 (-0.27 to 0.18) | - 0.09 (-0.34 to 0.16) | 0.99 | 0.97 | 2.2 | 3.8 |
| MPG | - 0.02 (-0.14 to 0.11) | - 0.03 (-0.21 to 0.15) | 0.98 | 0.97 | 4.1 | 4.6 |

*Mean bias (limits of agreement: ±1.96 standard deviations).

A_max_ = maximum pulmonary artery cross-sectional area；A_min_ = minimum pulmonary artery across-sectional area; AC = area change; AT = acceleration time; CoV = coefficient of variation; ET = ejection time; FPM = flow per min; ICC = intraclass correlation coefficient; MPG = mean pressure gradient; NFV = net flow volume; PPG = peak pressure gradient; PV = peak flow velocity; PWV = pulse wave velocity; RAC = relative area change.
